# Adverse effects of extracorporeal carbon dioxide removal (ECCO_2_R) for acute respiratory failure: a systematic review protocol

**DOI:** 10.1186/s13643-016-0270-0

**Published:** 2016-06-07

**Authors:** Zulian Liu, Rui V. Duarte, Sue Bayliss, George Bramley, Carole Cummins

**Affiliations:** Murray Learning Centre, Institute of Applied Health Research, University of Birmingham, Room 137, B15 2TT Birmingham, UK

**Keywords:** Extracorporeal membrane carbon dioxide removal, ECCO_2_R, Adverse effects, Hypercapnic respiratory failure, Lung transplantation

## Abstract

**Background:**

The extracorporeal membrane carbon dioxide removal (ECCO_2_R) system is primarily designed for the purpose of removing CO_2_ from the body for patients with potentially reversible severe acute hypercapnic respiratory failure or being considered for lung transplantation. Systematic reviews have focused on the effectiveness of ECCO_2_R. To the author’s best knowledge, this is the first systematic review to focus on the adverse effects of this procedure.

**Methods:**

We will conduct a systematic review of procedure-related adverse effects of ECCO_2_R systems. A high sensitivity search strategy will be employed in Cochrane Library, MEDLINE, EMBASE, Web of Science and product regulatory databases and ongoing trial registers to identify citations. Reference lists of relevant studies and grey literature will also be searched. Screening of the results will be performed by two reviewers independently using pre-defined inclusion and exclusion criteria. Clinical trials and observational studies will be included. Data will be extracted using a purposefully developed extraction form. Appropriateness for statistical pooling of the results will be determined and carried out if heterogeneity is low to moderate. The GRADE framework will be employed to grade the overall quality of the evidence.

**Discussion:**

In the UK, the current access to the use of ECCO_2_R is possible only with special arrangements for clinical governance, consent and for audit or research. Current evidence on ECCO_2_R suggests that there are a number of well-recognised complications which vary greatly across studies. This systematic review will consolidate the existing knowledge on adverse effects resulting from the use of ECCO_2_R.

**Systematic review registration:**

PROSPERO CRD42015023503.

**Electronic supplementary material:**

The online version of this article (doi:10.1186/s13643-016-0270-0) contains supplementary material, which is available to authorized users.

## Background

### Indications and current treatment

Acute respiratory failure is a suddenly occurring short-term life-threatening syndrome, in which the respiratory system fails in gas exchange function of oxygenation and/or carbon dioxide elimination, resulting in abnormally low levels of oxygen in the blood [hypoxia, i.e. arterial oxygen partial pressure (PaO_2_) <60 mmHg] and/or abnormally high carbon dioxide levels in the blood [hypercapnia, i.e. carbon dioxide partial pressure (PaCO_2_) >45 mmHg]. Classification depends on the absence (type 1) or presence (type 2) of hypercapnia. Type 1 respiratory failure is caused by conditions that damage the lung tissue, affecting the gas exchange in the lungs. Type 2 respiratory failure is caused by insufficient alveolar ventilation of the lungs to expel the carbon dioxide being produced. A common type of acute respiratory failure is acute respiratory distress syndrome (ARDS), which is a disease process resulting from a number of conditions including sepsis, pneumonia or chest trauma.

Conventional treatment for acute respiratory failure is mechanical ventilation. However, in some patients, hypoxia and/or hypercapnia are refractory to mechanical ventilation despite maximal tolerable ventilation settings (such as inspired oxygen concentration, airway pressures and tidal volume), which are associated with ventilation-induced lung injury. In the last few decades, extracorporeal membrane oxygenation (ECMO) has been occasionally used for carbon dioxide removal despite the primary purpose being oxygenation. In recent years, extracorporeal membrane carbon dioxide removal (ECCO_2_R) systems have been developed specifically for carbon dioxide removal in these patients to provide respiratory support for recovery, or in patients whose condition has not yet become refractory to facilitate protective ventilation, i.e. reduce the ventilation settings to decrease the risk of ventilation-induced lung injury [1, 2]. ECCO_2_R systems have also been used as a bridge to lung transplantation and ventilation weaning support [3–5]. UK surveys have shown that ECCO_2_R is used in a wider range of clinical conditions including chronic obstructive pulmonary disease (COPD), asthma and trauma with motivations that range from enabling lung protective ventilation to compassionate use [6].

### The technology

The ECCO_2_R system was developed from the principle of ECMO systems by underscoring the importance of carbon dioxide elimination rather than direct improvement of oxygenation in some patients. The circuit of the ECCO_2_R system can be set up in venovenous (VV) or arteriovenous (AV). For VV setup, a low flow pump is used to maintain a low extracorporeal flow rate using only 20–30 % of cardiac output. The ECCO_2_R system does not provide complete pulmonary function as it can achieve only limited oxygenation but provides predominantly carbon dioxide removal. As neither VV nor AV circuit allows full cardiopulmonary bypass, the system provides respiratory function but no cardiac support [1]. The types of ECCO2R system setup are summarised in Table 1.

**Table 1:** Types of ECCO_2_R system setup

	VV-ECCO_2_R	AV-ECCO_2_R
Pump	Yes	No
Extracorporeal flow rate	Low	Low
Gas exchange	Predominantly CO_2_ removal	Predominantly CO_2_ removal
Cardiac/respiratory support	Isolated respiratory failure	Isolated respiratory failure
Ventilation	Yes	Yes

### What the procedure involves

The procedure of extracorporeal respiratory support in the UK is currently restricted to highly specialised intensive care centres. The development of medical devices specifically to deliver ECCO_2_R rather than adaptation of ECMO circuits however allows wider dissemination of this technology. The initial cannulation is implanted by a surgeon under local anaesthesia, either percutaneously under ultrasound or fluoroscopy guidance, or via surgical cut. The cannulation sites are typically the femoral artery, femoral vein or internal jugular vein. AV cannulation needs a dual access system using an artery catheter and a venous catheter, while VV cannulation applies either a dual access system using two or three venous catheters, or a single access with a double lumen catheter. Extracorporeal blood flows via the cannulae continuously through the oxygenator, a low-resistance synthetic membrane device where oxygen is supplied as a “sweep gas” to remove CO_2_ that has diffused out of the blood, before returning to the patient’s blood circulation [1].

During the extracorporeal support, extracorporeal flow is monitored by an ultrasound device and can be modified by clamping the cannulae or adjusting the pump speed. Regular monitoring of arterial blood gases, cannulation sites and lower limb perfusion is also required. To reduce the risk of thrombus formation, the extracorporeal system is heparin-coated or, during the procedure, continuous heparin infusion is administered. Extracorporeal respiratory support is usually provided for periods ranging from days up to several weeks, depending on clinical need [2].

### Complications

The procedure can have mechanical- and/or patient-associated complications. Mechanical complications include circuit disruptions such as oxygenator failure, pump or heat exchanger malfunction, clots in the circuit plasma and problems with cannula. Patient-associated complications include system anticoagulation-related haemorrhage, cannulation site bleeding, haemolysis, heparin-induced thrombocytopenia, emboli, distal limb ischemia from arterial hypoperfusion and distal limb severe stasis (ischemia and oedema) from venous obstruction that may result in amputation [1, 2].

### Aim of the systematic review

This systematic review aims to identify adverse effects of the ECCO_2_R system for patients with potentially reversible severe acute hypercapnic respiratory failure or being considered for lung transplantation.

## Methods

This systematic review protocol follows the Preferred Reporting Items for Systematic Reviews and Meta-Analyses for Protocols (PRISMA-P) reporting guidelines [7]. A PRISMA-P checklist is included with this manuscript (see Additional file 1).

### Search strategy

Publicly available sources will be searched to identify studies and systematic reviews on the effectiveness and safety of ECCO_2_R. Electronic databases include the following: The Cochrane Library (Wiley) (including CDSR, DARE, HTA and CENTRAL), Medline (Ovid), Medline in Process (Ovid), EMBASE (Ovid), Science Citation Index (Web of Science), Conference Proceedings Citation Index (Web of Science) and ZETOC (British Library) will be searched from their commencement to date. An information specialist will develop the search strategy using a combination of both indexing and free text terms. This strategy will then be adapted to be run across each of the different databases. Other sources will also be searched including product regulatory databases and ongoing trial registers. Hand searching of reference lists of relevant studies will be carried out. Grey literature will also be searched. The search will be restricted to articles published in the English language. Literature search results will be uploaded to and managed using EndNote X7.0.1 software.

### Study selection

The selection criteria described in Table 2 below will be applied to the citations identified by the literature search. Two reviewers will independently screen the titles and abstracts of all retrieved citations and document the reasons for study exclusion. Where selection criteria could not be determined from the abstract, full paper of the citation will be retrieved. Full papers for studies which deemed potentially relevant by the screening will be retrieved. Any disagreements will be resolved by discussion and consensus between the reviewers; if consensus is not reached, a third reviewer will be consulted. If there is uncertainty whether a full text report meets all eligibility criteria, the authors will be contacted by email. In case of no response, the authors will be contacted at least twice over a 2-week period by email.

**Table 2:** Inclusion criteria for identification of relevant studies

Characteristic	Criteria
Population	Patients (including adults and children) with potentially reversible severe acute hypercapnic respiratory failure or being considered for lung transplantation
Intervention	ECCO_2_R system including VV and AV setups
Comparator	Because this systematic review focuses on adverse effects, any comparative and non-comparative studies will be included
Outcome measure	Any adverse effects including but not limited to arterial, venous and device thrombus formation; plasma leakage from gas exchange device; vascular access damage; infections; complications requiring surgery including lower limb amputation; gas embolism; haemolysis and heparin-induced thrombocytopenia
Study design	• Clinical trials and observational studies (including cohort studies, case-control studies, cross-sectional studies and case reports) • Systematic reviews, meta-analyses and narrative reviews will be included for identification of relevant primary studies
Language	Only English language literature will be included

### Quality assessment

Where appropriate, risk of bias of included studies will be assessed using the methods introduced in the Cochrane Handbook for Systematic Reviews of Interventions [8]. Two reviewers will conduct the assessment independently. Any disagreement will be resolved by discussion and consensus and if necessary consultation of a third reviewer.

### Data extraction

For each included study, data will be extracted by one reviewer and checked for accuracy by a second reviewer. Any disagreements will be resolved by discussion and if necessary consultation of a third reviewer. The following data fields will be included: (1) general information including study ID, author, year, journal, funding source, study design, setting; (2) recruitment details, sample size, demographics characteristics (age, gender) and baseline health data (diagnosis, co-morbidities); (3) type of ECCO_2_R system, ECCO_2_R system setup (i.e. VV and AV), diameter of cannulae; (4) indication, treatment duration, follow-up; (5) if applicable, quality assessment and methods of data analysis; (6) outcomes and definitions of outcomes. Study authors will not be contacted for missing data on adverse effects as this would be an onerous task considering the high volume of potentially relevant papers on this subject.

### Data synthesis and reporting

Studies will be grouped according to the type of study, indication, type of ECCO_2_R system, ECCO_2_R system setup (i.e. VV and AV) and diameter of cannulae, and data will be tabulated where appropriate. A narrative synthesis will be included as it is anticipated that this review will obtain information from a diverse body of evidence. Thus, a narrative synthesis may be necessary to provide potential explanations for contrasting findings observed in the literature. Appropriateness for statistical pooling will be determined initially based on an assessment of clinical and methodological heterogeneity of the studies. Statistical pooling of the results will be carried out using RevMan 5.2, if heterogeneity is low (0 to 40 %) to moderate (30 to 60 %) and data sufficient [8]. Dichotomous data will be expressed as risk ratio with 95 % confidence interval; mean differences or standardised mean difference with 95 % confidence interval will be used for continuous data. Intention-to-treat methods (i.e. according to group of allocation) will be adopted. Subgroup analysis and the *I*^2^ statistic will be applied to assess study heterogeneity where applicable. Publication bias will be assessed using a funnel plot, and sensitivity analysis on the basis of study quality will be conducted to explore the robustness of the meta-analysis if the data allow. Recommendations on testing for funnel plot asymmetry will be followed to determine whether it is appropriate to employ funnel plots and which tests to use based on between-study heterogeneity [9]. The GRADE framework will be employed to grade the overall quality of the evidence [10].

The findings from this systematic review will be reported according to the PRISMA harms checklist [11].

## Discussion

In June 2012, the National Institute for Health and Care Excellence (NICE) published Interventional Procedure Guidance 428 (IPG 428) on the safety and efficacy of ECCO_2_R for patients with severe acute respiratory failure [12]. The guidance recommends that ECCO_2_R should only be used in patients with potentially reversible hypercarbic respiratory failure or those being considered for lung transplantation; the procedure should only be undertaken with special arrangements for clinical governance, consent and for audit or research, because the evidence on the safety of ECCO_2_R showed a number of well-recognised complications, and evidence on its efficacy was limited in quality and quantity. NICE IPG 428 was based on a rapid review of the literature with an emphasis on good quality studies, without consideration for data from small case series and case reports [12]. The only two systematic reviews to date that assessed the efficacy and safety of ECCO_2_R did not specifically focus on the adverse effects of ECCO_2_R [13, 14]. In the Fitzgerald et al. review, studies reporting rarely occurring adverse effects may have been missed as the authors excluded studies with fewer than 10 patients (e.g. case report) [13]. Sklar et al. searched only MEDLINE and EMBASE and did not explore product regulatory databases that may include unpublished adverse effects [14]. Moreover, Sklar et al. focused on the use of ECCO_2_R for chronic obstructive pulmonary disease only. As previous systematic reviews have included only specific indications for ECCO_2_R [13, 14] and are unlikely to have identified all relevant reports of adverse effects, further evidence is required on adverse effects associated with ECCO_2_R used for any indication.

## Abbreviations

ARDS, acute respiratory distress syndrome; AV, arteriovenous; CO_2_, carbon dioxide; COPD, chronic obstructive pulmonary disease; ECCO_2_R, extracorporeal membrane carbon dioxide removal; ECMO, extracorporeal membrane oxygenation; GRADE, Grades of Recommendation, Assessment, Development and Evaluation; IPG, Interventional Procedure Guidance; NICE, National Institute for Health and Care Excellence; PaCO_2_, carbon dioxide partial pressure; PaO_2_, arterial oxygen partial pressure; PRISMA-P, Preferred Reporting Items for Systematic Reviews and Meta-Analyses for Protocols; VV, venovenous

## Additional file

Additional file 1: PRISMA-P checklist. (PDF 233 kb)
